# On Intuitionistic Fuzzy *β*-Almost Compactness and *β*-Nearly Compactness

**DOI:** 10.1155/2015/869740

**Published:** 2015-07-07

**Authors:** R. Renuka, V. Seenivasan

**Affiliations:** Department of Mathematics, University College of Engineering Panruti (A Constituent College of Anna University Chennai), Panruti, Tamil Nadu 607 106, India

## Abstract

The concept of intuitionistic fuzzy *β*-almost compactness and intuitionistic fuzzy *β*-nearly compactness in intuitionistic fuzzy topological spaces is introduced and studied. Besides giving characterizations of these spaces, we study some of their properties. Also, we investigate the behavior of intuitionistic fuzzy *β*-compactness, intuitionistic fuzzy *β*-almost compactness, and intuitionistic fuzzy *β*-nearly compactness under several types of intuitionistic fuzzy continuous mappings.

## 1. Introduction

As a generalization of fuzzy sets introduced by Zadeh [1], the concept of intuitionistic fuzzy set was introduced by Atanassov [2]. Coker [3] introduced intuitionistic fuzzy topological spaces. Later on, Coker [3] introduced fuzzy compactness in intuitionistic fuzzy topological spaces. In [4], the concept of intuitionistic fuzzy *β*-compactness was defined. In this paper some properties of intuitionistic fuzzy *β*-compactness were investigated. We use the finite intersection property to give characterization of the intuitionistic fuzzy *β*-compact spaces. Also we introduce intuitionistic fuzzy *β*-almost compactness and intuitionistic fuzzy *β*-nearly compactness and established the relationships between these types of compactness.

## 2. Preliminaries

**Definition 1 (see [2]).**

Let *X* be a nonempty fixed set and *I* the closed interval [0,1]. An intuitionistic fuzzy set (IFS) A is an object of the following form:

(1) $$\begin{array}{c} A=\left\{\;\langle \;x,\mu_{A}\left(\;x\;\right),\nu_{A}\left(\;x\;\right)\;\rangle ;\;\;x\in X\;\right\}, \end{array}$$

where the mappings *μ*_*A*_(*x*) : *X* → *I* and *ν*_*A*_(*x*) : *X* → *I* denote the degree of membership, namely, *μ*_*A*_(*x*), and the degree of nonmembership, namely, *ν*_*A*_(*x*), for each element *x* ∈ *X* to the set *A*, respectively, and 0 ≤ *μ*_*A*_(*x*) + *ν*_*A*_(*x*) ≤ 1 for each *x* ∈ *X*.

**Definition 2 (see [2]).**

Let *A* and *B* be IFSs of the forms *A* = {〈*x*, *μ*_*A*_(*x*), *ν*_*A*_(*x*)〉; *x* ∈ *X*} and *B* = {〈*x*, *μ*_*B*_(*x*), *ν*_*B*_(*x*)〉; *x* ∈ *X*}. Then

- *A*⊆*B* if and only if *μ*_*A*_(*x*) ≤ *μ*_*B*_(*x*) and *ν*_*A*_(*x*) ≥ *ν*_*B*_(*x*);
- $\bar{A}\;\;(or\;\;A^{c})=\{\langle x,\nu_{A}(x),\mu_{A}(x)\rangle ;\;\;x\in X\}$;
- *A*∩*B* = {〈*x*, *μ*_*A*_(*x*)∧*μ*_*B*_(*x*), *ν*_*A*_(*x*)∨*ν*_*B*_(*x*)〉; *x* ∈ *X*};
- *A* ∪ *B* = {〈*x*, *μ*_*A*_(*x*)∨*μ*_*B*_(*x*), *ν*_*A*_(*x*)∧*ν*_*B*_(*x*)〉; *x* ∈ *X*}.

We will use the notation *A* = {〈*x*, *μ*_*A*_, *ν*_*A*_〉; *x* ∈ *X*} instead of *A* = {〈*x*, *μ*_*A*_(*x*), *ν*_*A*_(*x*)〉; *x* ∈ *X*}.

**Definition 3 (see [2]).**

Consider 0_~_ = {〈*x*, 0,1〉; *x* ∈ *X*} and 1_~_ = {〈*x*, 1,0〉; *x* ∈ *X*}.

Let *α*, *β* ∈ [0,1] such that *α* + *β* ≤ 1. An intuitionistic fuzzy point (IFP) *p*_(*α*, *β*)_ is intuitionistic fuzzy set defined by

(2) $$\begin{array}{c} p_{\left(\alpha ,\beta \right)}\left(\;x\;\right)=\left\{\begin{array}{cc} \left(\alpha ,\beta \right) & \text{if }x=p,\\ \left(0,1\right) & \text{otherwise.} \end{array}\right. \end{array}$$

**Definition 4 (see [3]).**

An intuitionistic fuzzy topology (IFT) in Coker's sense on a nonempty set *X* is a family *τ* of intuitionistic fuzzy sets in *X* satisfying the following axioms:

- 0_~_, 1_~_ ∈ *τ*;
- *G*_1_∩*G*_2_ ∈ *τ*, for any *G*_1_, *G*_2_ ∈ *τ*;
- ∪⁡*G*_*i*_ ∈ *τ* for any arbitrary family {*G*_*i*_; *i* ∈ *J*}⊆*τ*.

In this paper by (*X*, *τ*) and (*Y*, *σ*) or simply by *X* and *Y*, we will denote the intuitionistic fuzzy topological spaces (IFTSs). Each IFS which belongs to *τ* is called an intuitionistic fuzzy open set (IFOS) in *X*. The complement $\overset{-}{A}$ of an IFOS A in *X* is called an intuitionistic fuzzy closed set (IFCS) in *X*.

Let *X* and *Y* be two nonempty sets and let *f* : (*X*, *τ*)→(*Y*, *σ*) be a function.

If *B* = {〈*y*, *μ*_*B*_(*y*), *ν*_*B*_(*y*)〉; *y* ∈ *Y*} is an IFS in *Y*, then the preimage of *B* under *f* is denoted and defined by *f* ^−1^(*B*) = {〈*x*, *f* ^−1^(*μ*_*B*_(*x*)), *f* ^−1^(*ν*_*B*_(*x*))〉; *x* ∈ *X*}. Since *μ*_*B*_(*x*), *ν*_*B*_(*x*) are fuzzy sets, we explain that *f* ^−1^(*μ*_*B*_(*x*)) = *μ*_*B*_(*x*)(*f*(*x*)) and *f* ^−1^(*ν*_*B*_(*x*)) = *ν*_*B*_(*x*)(*f*(*x*)).

**Definition 5 (see [5]).**

Let *p*_(*α*,*β*)_ be an IFP in IFTS *X*. An IFS *A* in *X* is called an intuitionistic fuzzy neighborhood (IFN) of *p*_(*α*,*β*)_ if there exists an IFOS *B* in *X* such that *p*_(*α*,*β*)_∈*B*⊆*A*.

**Definition 6 (see [3]).**

Let (*X*, *τ*) be an IFTS and let *A* = {〈*x*, *μ*_*A*_(*x*), *ν*_*A*_(*x*)〉; *x* ∈ *X*} be an IFS in *X*. Then the intuitionistic fuzzy closure and intuitionistic fuzzy interior of *A* are defined by

- cl (*A*) = ∩{*C* : *C*  is  an  IFCS  in  *X*  and  *C*⊇*A*};
- int (*A*) = ∪{*D* : *D*  is  an  IFOS  in  *X*  and  *D*⊆*A*}.

It can be also shown that cl (*A*) is an IFCS, int (*A*) is an IFOS in *X*, and *A* is an IFCS in *X* if and only if cl (*A*) = *A*; *A* is an IFOS in *X* if and only if int (*A*) = *A*.

**Proposition 7 (see [3]).**

Let (*X*, *τ*) be an IFTS and let *A* and *B* be IFSs in *X*. Then the following properties hold:

- $cl\;\bar{A}=\bar{\left(int\;(A)\right)}$, $int\;\bar{\left(A\right)}=\bar{\left(cl\;(A)\right)}$;
- int (*A*)⊆*A*⊆cl (*A*).

**Definition 8 (see [6]).**

An IFS *A* in an IFTS *X* is called an intuitionistic fuzzy *β*-open set (IF*β*OS) if *A*⊆cl(int(cl*A*)). The complement of an IF*β*OS A in IFTS *X* is called an intuitionistic fuzzy *β*-closed set (IF*β*CS) in *X*.

**Definition 9 (see [6]).**

Let *f* be a mapping from an IFTS *X* into an IFTS *Y*. The mapping *f* is called

- intuitionistic fuzzy continuous if and only if *f* ^−1^(*B*) is an IFOS in *X*, for each IFOS *B* in *Y*;
- intuitionistic fuzzy *β*-continuous if and only if *f* ^−1^(*B*) is an IF*β*OS in *X*, for each IFOS *B* in *Y*.

**Definition 10 (see [4]).**

Let (*X*, *τ*) be an IFTS and let *A* = {〈*x*, *μ*_*A*_(*x*), *ν*_*A*_(*x*)〉; *x* ∈ *X*} be an IFS in *X*. Then the intuitionistic fuzzy *β*-closure and intuitionistic fuzzy *β*-interior of *A* are defined by

- *β*cl⁡(*A*) = ∩{*C* : *C*  is  an  IF*β*CS  in  *X*  and  *C*⊇*A*};
- *β*int⁡(*A*) = ∪{*D* : *D*  is  an  IF*β*OS  in  *X*  and  *D*⊆*A*}.

**Definition 11 (see [7]).**

A fuzzy function *f* : *X* → *Y* is called fuzzy *β*-irresolute if inverse image of each fuzzy *β*-open set is fuzzy *β*-open.

**Definition 12 (see [4]).**

A function *f* : (*X*, *τ*)→(*Y*, *σ*) from an intuitionistic fuzzy topological space (*X*, *τ*) to another intuitionistic fuzzy topological space (*Y*, *σ*) is said to be intuitionistic fuzzy *β*-irresolute if *f* ^−1^(*B*) is an IF*β*OS in (*X*, *τ*) for each IF*β*OS *B* in (*Y*, *σ*).

**Definition 13 (see [3, 4]).**

Let *X* be an IFTS. A family of {〈*x*, *μ*_*G*_*i*__(*x*), *ν*_*G*_*i*__(*x*)〉; *i* ∈ *J*} intuitionistic fuzzy open sets (intuitionistic fuzzy *β*-open sets) in *X* satisfies the condition 1_~_ = ∪{〈*x*, *μ*_*G*_*i*__(*x*), *ν*_*G*_*i*__(*x*)〉; *i* ∈ *J*} which is called an intuitionistic fuzzy open (intuitionistic fuzzy *β*-open) cover of *X*. A finite subfamily of an intuitionistic fuzzy open (intuitionistic fuzzy *β*-open) cover {〈*x*, *μ*_*G*_*i*__(*x*), *ν*_*G*_*i*__(*x*)〉; *i* ∈ *J*} of *X* which is also an intuitionistic fuzzy open (intuitionistic fuzzy *β*-open) cover of *X* is called a finite subcover of {〈*x*, *μ*_*G*_*i*__(*x*), *ν*_*G*_*i*__(*x*)〉; *i* ∈ *j*}.

**Definition 14 (see [3]).**

An IFTS *X* is called intuitionistic fuzzy compact if each fuzzy open cover of *X* has a finite subcover for *X*.

**Definition 15 (see [8]).**

An IFTS *X* is called intuitionistic fuzzy almost compact (IF almost compact) if, for every IF open cover {*U*_*j*_ : *j* ∈ *J*} of *X*, there exists a finite subfamily *J*_0_ ⊂ *J* such that *X* = ∪{cl⁡(*U*_*j*_) : *j* ∈ *J*_0_}.

**Definition 16 (see [4]).**

An IFTS *X* is said to be intuitionistic fuzzy *β*-compact (IF *β*-compact) if every IF *β*-open cover of *X* has a finite subcover.

**Definition 17 (see [6]).**

Let (*X*, *τ*) and (*Y*, *σ*) be two IFTSs. A function *f* : *X* → *Y* is said to be intuitionistic fuzzy weakly continuous (IF weakly continuous) if, for each IFOS *V* in *Y*, *f* ^−1^(*V*)⊆int (*f* ^−1^(cl⁡*V*)).

## 3. Intuitionistic Fuzzy *β*-Compactness

**Definition 18 .**

An IFTS *B* of (*X*, *τ*) is said to be IF *β*-compact relative to *X* if, for every collection {*A*_*i*_; *i* ∈ *I*} of IF *β*-open subsets of *X* such that *B*⊆∪{*A*_*i*_; *i* ∈ *I*}, there exists a finite subset *I*_0_ of *I* such that *B*⊆∪{*A*_*i*_; *i* ∈ *I*_0_}.

**Definition 19 .**

An IFTS *B* of (*X*, *τ*) is said to be IF *β*-compact if it is IF *β*-compact as a subspace of *X*.

**Definition 20 .**

A family of IF *β*-closed sets {*A*_*i*_; *i* ∈ *I*} has the finite intersection property (in short FIP) if, for any subset *I*_0_ of *I*, ∩_*i*∈*I*_0__ *A*_*i*_ ≠ 0_~_.

**Theorem 21 .**

For an IFTS *X* the following statements are equivalent.

- *X* is IF *β*-compact.
- Any family of IF *β*-closed subsets of *X* satisfying the FIP has a nonempty intersection.

**Proof**

Let *X* be IF *β*-compact space and let {*A*_*i*_; *i* ∈ *I*} be a family of IF *β*-closed subsets of *X* satisfying the FIP. Suppose ∩_*i*∈*I*_ *A*_*i*_ = 0_~_. Then $\cup_{i\in I}\bar{A_{i}}=1_{\sim }$. Since $\left\{\bar{A_{i}};\;\;i\in I\right\}$ is a collection of IF *β*-open sets cover *X*, then from IF *β*-compactness of *X* there exists a finite subset *I*_0_ of *I* such that $\cup_{i\in I_{0}}\bar{A_{i}}=1_{\sim }$. Then ∩_*i*∈*I*_0__ *A*_*i*_ = 0_~_ which gives a contradiction and therefore ∩_*i*∈*I*_ *A*_*i*_ ≠ 0_~_. Thus (i)⇒(ii).

Let {*A*_*i*_; *i* ∈ *I*} be a family of IF *β*-open sets cover *X*. Suppose that for any finite subset *I*_0_ of *I* we have ∪_*i*∈*I*_0__ *A*_*i*_ ≠ 1_~_. Then $\cap_{i\in I_{0}}\bar{A_{i}}\neq 0_{\sim }$. Hence $\left\{\bar{A_{i}};\;\;i\in I\right\}$ satisfies the FIP. Then, by hypothesis, we have $\cap_{i\in I}\bar{A_{i}}\neq 0_{\sim }$ which implies that ∪_*i*∈*I*_ *A*_*i*_ ≠ 1_~_ and contradicts that {*A*_*i*_; *i* ∈ *I*} is an IF *β*-open cover of *X*. Hence our assumption ∪_*i*∈*I*_0__ *A*_*i*_ ≠ 1_~_ is wrong. Thus ∪_*i*∈*I*_0__ *A*_*i*_ = 1_~_ which implies that *X* is IF *β*-compact. Thus (ii)⇒(i).

**Theorem 22 .**

An intuitionistic fuzzy *β*-closed subset of an intuitionistic fuzzy *β*-compact space is intuitionistic fuzzy *β*-compact relative to *X*.

**Proof**

Let *A* be an IF *β*-closed subset of *X*. Let {*G*_*i*_; *i* ∈ *I*} be cover of *A* by IF *β*-open sets in *X*. Then the family $\{G_{i};\;\;i\in I\}\cup \bar{A}$ is an IF *β*-open cover of *X*. Since *X* is IF *β*-compact, there is a finite subfamily {*G*_1_, *G*_2_,…, *G*_*n*_} of IF *β*-open cover, which also covers *X*. If this cover contains $\bar{A}$, we discard it. Otherwise leave the subcover as it is. Thus we obtained a finite IF *β*-open subcover of *A*. So *A* is IF *β*-compact relative to *X*.

**Theorem 23 .**

Let (*X*, *τ*) and (*Y*, *σ*) be intuitionistic fuzzy topological spaces and let *f* : (*X*, *τ*)→(*Y*, *σ*) be intuitionistic fuzzy *β*-irresolute, surjective mapping. If (*X*, *τ*) is IF *β*-compact space then so is (*Y*, *σ*).

**Proof**

Let *f* : (*X*, *τ*)→(*Y*, *σ*) be intuitionistic fuzzy *β*-irresolute mapping of an intuitionistic fuzzy *β*-compact space (*X*, *τ*) onto an IFTS (*Y*, *σ*). Let {*A*_*i*_ : *i* ∈ *I*} be any intuitionistic fuzzy *β*-open cover of (*Y*, *σ*). Then {*f* ^−1^(*A*_*i*_) : *i* ∈ *I*} is collection of intuitionistic fuzzy *β*-open sets which covers *X*. Since *X* is intuitionistic fuzzy *β*-compact, there exists a finite subset *I*_0_ of *I* such that subfamily {*f* ^−1^(*A*_*i*_); *i* ∈ *I*_0_} of {*f* ^−1^(*A*_*i*_):  *i* ∈ *I*} covers *X*. It follows that {*A*_*i*_; *i* ∈ *I*_0_} is a finite subfamily of {*A*_*i*_ : *i* ∈ *I*} which covers *Y*. Hence *Y* is intuitionistic fuzzy *β*-compact.

**Theorem 24 .**

Let (*X*, *τ*) and (*Y*, *σ*) be intuitionistic fuzzy topological spaces and let *f* : (*X*, *τ*)→(*Y*, *σ*) be intuitionistic fuzzy *β*-irresolute mapping. If *A* is IF *β*-compact relative to *X* then *f*(*A*) is IF *β*-compact relative to *Y*.

**Proof**

Let {*A*_*i*_ : *i* ∈ *I*} be a family of IF *β*-open cover of *Y* such that *f*(*A*)⊆∪_*i*∈*I*_ *A*_*i*_. Then *A*⊆*f* ^−1^(*f*(*A*))⊆*f* ^−1^(∪_*i*∈*I*_ *A*_*i*_) = ∪_*i*∈*I*_ *f* ^−1^(*A*_*i*_). Since *f* is IF *β*-irresolute, *f* ^−1^(*A*_*i*_) is IF *β*-open cover of *X*. And *A* is IF *β*-compact in (*X*, *τ*); there exists a finite subset *I*_0_ of *I* such that *A*⊆∪_*i*∈*I*_0__ *f* ^−1^(*A*_*i*_). Hence *f*(*A*)⊆*f*(∪_*i*∈*I*_0__ *f* ^−1^(*A*_*i*_)) = ∪_*i*∈*I*_0__ *f*(*f* ^−1^(*A*_*i*_))⊆∪_*i*∈*I*_0__(*A*_*i*_). Thus *f*(*A*) is IF *β*-compact relative to *Y*.

**Theorem 25 .**

An IF *β*-continuous image of IF *β*-compact space is IF compact.

**Proof**

Let *f* : (*X*, *τ*)→(*Y*, *σ*) be an IF *β*-continuous from an IF *β*-compact space *X* onto IFTS *Y*. Let {*A*_*i*_ : *i* ∈ *I*} be IF open cover of *Y*. Then {*f* ^−1^(*A*_*i*_); *i* ∈ *I*} is IF *β*-open cover of *X*. Since *X* is IF *β*-compact, there exists finite subset *I*_0_ of *I* such that finite family {*f* ^−1^(*A*_*i*_); *i* ∈ *I*_0_} covers *X*. Since *f* is onto, {*A*_*i*_ : *i* ∈ *I*_0_} is a finite cover of *Y*. Hence *Y* is IF compact.

**Definition 26 .**

Let (*X*, *τ*) and (*Y*, *σ*) be two intuitionistic fuzzy topological spaces. A mapping *f* : *X* → *Y* is said to be intuitionistic fuzzy strongly *β*-open if *f*(*V*) is IF*β*OS of *Y* for every IF*β*OS *V* of *X*.

**Theorem 27 .**

Let *f* : (*X*, *τ*)→(*Y*, *σ*) be an IF strongly *β*-open, bijective function and *Y* is IF *β*-compact; then *X* is IF *β*-compact.

**Proof**

Let {*A*_*i*_ : *i* ∈ *I*} be IF *β*-open cover of *X*, and then {*f*(*A*_*i*_) : *i* ∈ *I*} is IF *β*-open cover of *Y*. Since *Y* is IF *β*-compact, there is a finite subset *I*_0_ of *I* such that finite family {*f*(*A*_*i*_) : *i* ∈ *I*_0_} covers *Y*. But 1_~*X*_ = *f* ^−1^(1_~*Y*_) = *f* ^−1^ *f*(∪_*i*∈*I*_0__(*A*_*i*_)) = ∪_*i*∈*I*_0__ *A*_*i*_ and therefore *X* is IF *β*-compact.

## 4. Intuitionistic Fuzzy *β*-Almost Compactness and Intuitionistic Fuzzy *β*-Nearly Compactness

In this section we investigate the relationships between IF *β*-compactness, IF *β*-almost compactness, and IF *β*-nearly compactness.

**Definition 28 .**

An IFTS (*X*, *τ*) is said to be IF *β*-almost compactness if and only if, for every family of IF *β*-open cover {*A*_*i*_ : *i* ∈ *I*} of *X*, there exists a finite subset *I*_0_ of *I* such that ∪_*i*∈*I*_0__ *β*cl⁡*A*_*i*_ = 1_~_.

**Definition 29 .**

An IFTS (*X*, *τ*) is said to be IF *β*-nearly compactness if and only if, for every family of IF *β*-open cover {*A*_*i*_ : *i* ∈ *I*} of *X*, there exists a finite subset *I*_0_ of *I* such that ∪_*i*∈*I*_0__ *β*int⁡(*β*cl⁡*A*_*i*_) = 1_~_.

**Definition 30 .**

An IFTS (*X*, *τ*) is said to be IF *β*-regular if, for each IF *β*-open set *A* ∈ *X*, *A* = ∪{*A*_*i*_ ∈ *I* ^*X*^∣*A*_*i*_  is  IF  *β*-open, *β*cl⁡*A*_*i*_⊆*A*}.

**Theorem 31 .**

Let (*X*, *τ*) be IFTS. Then IF *β*-compactness implies IF *β*-nearly compactness which implies IF *β*-almost compactness.

**Proof**

Let (*X*, *τ*) be IF *β*-compact. Then for every IF *β*-open cover {*A*_*i*_ : *i* ∈ *I*} of *X*, there exists a finite subset ∪_*i*∈*I*_0__ *A*_*i*_ = 1_~_. Since *A*_*i*_ is an IF*β*OS, for each *i* ∈ *I*, *A*_*i*_ = *β*int⁡(*A*_*i*_) for each *i* ∈ *I*. *A*_*i*_ = *β*int⁡*A*_*i*_⊆*β*int⁡(*β* cl ⁡*A*_*i*_) for each *i* ∈ *I*. Here 1_~_ = ∪_*i*∈*I*_0__ *A*_*i*_ = ∪_*i*∈*I*_0__ *β*int⁡*A*_*i*_⊆∪_*i*∈*I*_0__ *β*int⁡(*β* cl ⁡*A*_*i*_). Thus ∪_*i*∈*I*_0__ *β*int⁡(*β*cl⁡*A*_*i*_) = 1_~_ which implies that (*X*, *τ*) is IF *β*-nearly compactness. Now let (*X*, *τ*) be IF *β*-nearly compact. Then for every IF *β*-open cover {*A*_*i*_ : *i* ∈ *I*} of *X*, there exists a finite subset ∪_*i*∈*I*_0__ *β*int⁡(*β*cl⁡*A*_*i*_) = 1_~_. Since *β*int⁡(*β*cl⁡*A*_*i*_)⊆*β*cl⁡*A*_*i*_ for each *i* ∈ *I*_0_, 1_~_ = ∪_*i*∈*I*_0__ *β*int⁡(*β*cl⁡*A*_*i*_)⊆∪_*i*∈*I*_0__ *β*cl⁡*A*_*i*_. Thus ∪_*i*∈*I*_0__ *β*cl⁡*A*_*i*_ = 1_~_. Hence (*X*, *τ*) is IF *β*-almost compact.

**Remark 32 .**

Converse implications in theorem are not true in general.

**Example 33 .**

Let *X* be a nonempty set. Then (*X*, *τ*) is IFTS, where *τ* = {0_~_, 1_~_, *A*_*n*_}, *n* ∈ *N*, where *A*_*n*_ : *X* → [0,1] is defined by *A*_*n*_ = {〈*x*, 1 − 1/*n*, 1/*n*〉; *x* ∈ *X*}, *n* ∈ *N*. The collection {*A*_*n*_ : *n* ∈ *N*} is IF *β*-open cover of *X*. But no finite subset of {*A*_*n*_ : *n* ∈ *N*} covers *X*. Hence *X* is* not IF β-compact*. But *β*cl⁡*A*_*n*_ = 1_~_ for *n* ≥ 3. Thus there exists a finite subfamily {*A*_*n*_ : *n* ∈ *N*_0_} for *N*_0_⊆*N* such that ∪_*n*∈*N*_0__ *β*cl⁡*A*_*n*_ = 1_~_. Thus *X* is* IF β-almost compactness*. Also *β*int⁡(*β*cl⁡*A*_*n*_) = *β*int⁡ (1_~_) = 1_~_ for *n* ≥ 3. Thus there exists a finite subfamily {*A*_*n*_ : *n* ∈ *N*_0_} for *N*_0_⊆*N* such that ∪_*n*∈*N*_0__ *β*int⁡*β*cl⁡*A*_*n*_ = 1_~_. Thus *X* is* IF β-nearly compactness*.

**Theorem 34 .**

Let (*X*, *τ*) be IFTS. If (*X*, *τ*) is IF *β*-almost compact and IF *β*-regular then (*X*, *τ*) is IF *β*-compact.

**Proof**

Let {*A*_*i*_ : *i* ∈ *I*} be IF *β*-open cover of *X* such that ∪_*i*∈*I*_ *A*_*i*_ = 1_~_. Since (*X*, *τ*) is IF *β*-regular, *A*_*i*_ = ∪{*B*_*i*_ ∈ *I* ^*X*^∣*B*_*i*_  is  IF  *β*-open, *β*cl⁡ *B*_*i*_⊆*A*_*i*_} for each *i* ∈ *I*. Since 1_~_ = ∪_*i*∈*I*_(∪_*i*∈*I*_ *B*_*i*_) and (*X*, *τ*) is IF *β*-almost compact there exists a finite set *I*_0_ of *I* such that ∪_*i*∈*I*_0__ *β*cl⁡*B*_*i*_ = 1_~_. But *β*cl⁡(*B*_*i*_)⊆*A*_*i*_  (*β*int⁡(*β*cl⁡*B*_*i*_)⊆*β*cl⁡(*B*_*i*_)). We have ∪_*i*∈*I*_0__ *A*_*i*_⊇∪_*i*∈*I*_0__ *β*cl⁡*B*_*i*_ = 1_~_. Thus, ∪_*i*∈*I*_0__ *A*_*i*_ = 1_~_. Hence (*X*, *τ*) is IF *β*-compact.

**Theorem 35 .**

Let (*X*, *τ*) be IFTS. If (*X*, *τ*) is IF *β*-nearly compact and IF *β*-regular then (*X*, *τ*) is IF *β*-compact.

**Proof**

Let {*A*_*i*_ : *i* ∈ *I*} be IF *β*-open cover of *X* such that ∪_*i*∈*I*_ *A*_*i*_ = 1_~_. Since (*X*, *τ*) is IF *β*-regular, *A*_*i*_ = ∪{*B*_*i*_ ∈ *I* ^*X*^∣*B*_*i*_  is  IF  *β*-open, *β* cl⁡ *B*_*i*_⊆*A*_*i*_} for each *i* ∈ *I*. Since 1_~_ = ∪_*i*∈*I*_(∪_*i*∈*I*_ *B*_*i*_) and (*X*, *τ*) is IF *β*-nearly compact there exists a finite set *I*_0_ of *I* such that ∪_*i*∈*I*_0__ *β*int⁡(*β*cl⁡⁡*B*_*i*_) = 1_~_. But *β*int ⁡(*β*cl⁡*B*_*i*_)⊆*β*cl⁡(*B*_*i*_)⊆*A*_*i*_. We have ∪_*i*∈*I*_0__ *A*_*i*_⊇∪_*i*∈*I*_0__ *β*cl⁡*B*_*i*_⊇∪_*i*∈*I*_0__ *β*int⁡(*β*cl⁡*B*_*i*_) = 1_~_. Thus, ∪_*i*∈*I*_0__ *A*_*i*_ = 1_~_. Hence (*X*, *τ*) is IF *β*-compact.

**Theorem 36 .**

An IFTS (*X*, *τ*) is IF *β*-almost compact, if and only if, for every family {*A*_*i*_ : *i* ∈ *I*} of IF *β*-open sets having the FIP, ∩_*i*∈*I*_ *β*cl⁡*A*_*i*_ ≠ 0_~_.

**Proof**

Let {*A*_*i*_ : *i* ∈ *I*} be a family of IF *β*-open sets having the FIP. Suppose that ∩_*i*∈*I*_ *β*cl⁡*A*_*i*_ = 0_~_ and then $\cup_{i\in I}\bar{\beta \mathrm{cl}A_{i}}=\cup_{i\in I}\beta \mathrm{int}\bar{A_{i}}=1_{\sim }$. Since (*X*, *τ*) is IF *β*-almost compact, there exists a finite subset *I*_0_ of *I* such that $\cup_{i\in I_{0}}\beta \mathrm{cl}\left(\beta \mathrm{int}\bar{A_{i}}\right)=1_{\sim }$. This implies that $\cup_{i\in I_{0}}\beta \mathrm{cl}\left(\beta \mathrm{int}\bar{A_{i}}\right)=\cup_{i\in I_{0}}\beta \mathrm{cl}\left(\bar{\beta \mathrm{cl}A_{i}}\right)=1_{\sim }$. Thus, ∩_*i*∈*I*_0__ *β*int⁡(*β* cl *A*_*i*_) = 0_~_. But *A*_*i*_ = *β*⁡int⁡*A*_*i*_⊆*β*int ⁡(*β*cl⁡*A*_*i*_). This implies that ∩_*i*∈*I*_0__ *A*_*i*_ = 0_~_ which is in contradiction with FIP of the family. Conversely, let {*A*_*i*_ : *i* ∈ *I*} be a family of IF *β*-open sets such that ∪_*i*∈*I*_ *A*_*i*_ = 1_~_. Suppose that there exists no finite subset *I*_0_ of *I* such that ∪_*i*∈*I*_0__ *β*cl⁡*A*_*i*_ = 1_~_. Since $\left\{\bar{\beta \mathrm{cl}A_{i}};\;\;i\in I\right\}$ has the FIP then $\cap_{i\in I}\beta \mathrm{cl}\left(\bar{\beta \mathrm{cl}A_{i}}\right)\neq 0_{\sim }$. This implies $\cup_{i\in I}\bar{\beta \mathrm{cl}\;\left(\bar{\beta \mathrm{cl}A_{i}}\right)}\neq 1_{\sim }$. Hence ${\underset{}{\cup }}_{i\in I}\beta \mathrm{int}\;(\beta \mathrm{cl}A_{i})\neq 1_{\sim }$. Since *A*_*i*_⊆*β*int⁡ (*β*cl⁡*A*_*i*_) for each *i* ∈ *I*, ∪_*i*∈*I*_ *A*_*i*_ ≠ 1_~_ which is in contradiction with ∪_*i*∈*I*_ *A*_*i*_ = 1_~_.

**Theorem 37 .**

Let (*X*, *τ*) and (*Y*, *σ*) be IFTS and let *f* : (*X*, *τ*)→(*Y*, *σ*) be intuitionistic fuzzy *β*-irresolute, surjective mapping. If (*X*, *τ*) is IF *β*-almost compact space then so is (*Y*, *σ*).

**Proof**

Let *f* : (*X*, *τ*)→(*Y*, *σ*) be intuitionistic fuzzy *β*-irresolute mapping of an intuitionistic fuzzy *β*-compact space (*X*, *τ*) onto an IFTS (*Y*, *σ*). Let {*A*_*i*_ : *i* ∈ *I*} be any intuitionistic fuzzy *β*-open cover of (*Y*, *σ*). Then {*f* ^−1^(*A*_*i*_) : *i* ∈ *I*} is an intuitionistic fuzzy *β*-open cover of *X*. Since *X* is intuitionistic fuzzy *β*-almost compact, there exists a finite subset *I*_0_ of *I* such that ∪_*i*∈*I*_0__ *β*cl⁡ (*f* ^−1^(*A*_*i*_)) = 1_~*X*_. And *f*(1_~*X*_) = *f*(∪_*i*∈*I*_0__ *β*cl⁡(*f* ^−1^(*A*_*i*_))) = ∪_*i*∈*I*_0__ *f*(*β*cl⁡(*f* ^−1^(*A*_*i*_))) = 1_~*Y*_. But *β*cl⁡ (*f* ^−1^(*A*_*i*_))⊆*f* ^−1^(*β*cl *A*_*i*_) and from the surjectivity of *f*, *f*(*β*cl (*f* ^−1^(*A*_*i*_)))⊆*f*(*f* ^−1^(*β*cl⁡*A*_*i*_)) = *β*cl⁡*A*_*i*_. So ∪_*i*∈*I*_0__ *β*cl⁡*A*_*i*_⊇∪_*i*∈*I*_0__ *f*(*β*cl⁡(*f* ^−1^(*A*_*i*_))) = 1_~*Y*_. Thus ∪_*i*∈*I*__0___ *β*cl⁡*A*_*i*_ = 1_~*Y*_. Hence (*Y*, *σ*) is IF *β*-almost compact.

**Theorem 38 .**

Let (*X*, *τ*) and (*Y*, *σ*) be intuitionistic fuzzy topological spaces and let *f* : (*X*, *τ*)→(*Y*, *σ*) be intuitionistic fuzzy *β*-continuous, surjective mapping. If (*X*, *τ*) is IF *β*-almost compact space then (*Y*, *σ*) is IF almost compact.

**Proof**

Let {*A*_*i*_ : *i* ∈ *I*} be any intuitionistic fuzzy open cover of (*Y*, *σ*). Then {*f* ^−1^(*A*_*i*_) : *i* ∈ *I*} is an intuitionistic fuzzy *β*-open cover of *X*. Since *X* is intuitionistic fuzzy *β*-almost compact, there exists a finite subset *I*_0_ of *I* such that ∪_*i*∈*I*_0__ *β*cl⁡(*f* ^−1^(*A*_*i*_)) = 1_~*X*_. And from the surjectivity of *f*, 1_~*Y*_ = *f*(1_~*X*_) = *f*(∪_*i*∈*I*_0__ *β*cl⁡(*f* ^−1^(*A*_*i*_)))⊆∪_*i*∈*I*_0__ *f*(*β*cl⁡(*f* ^−1^(*A*_*i*_)))∪_*i*∈*I*_0__ *β*cl⁡*f*(*f* ^−1^(*A*_*i*_))⊆∪_*i*∈*I*_0__cl⁡*f*(*f* ^−1^(*A*_*i*_))⊆∪_*i*∈*I*_0__cl ⁡*A*_*i*_ which implies that ∪_*i*∈*I*_0__cl⁡*A*_*i*_ = 1_~*Y*_. Hence (*Y*, *σ*) is IF almost compact.

**Definition 39 .**

Let (*X*, *τ*) and (*Y*, *σ*) be two intuitionistic fuzzy topological spaces. A function *f* : *X* → *Y* is said to be intuitionistic fuzzy *β*-weakly continuous (IF *β*-weakly continuous) if, for each IF*β*OS *V* in *Y*, *f* ^−1^(*V*)⊆*β*int⁡(*f* ^−1^(*β*cl⁡*V*)).

**Theorem 40 .**

A mapping *f* from an IFTS (*X*, *τ*) to an IFTS (*Y*, *σ*) is IF strongly *β*-open if and only if *f*(*β*int⁡*V*)⊆*β*int⁡*f*(*V*).

**Proof**

If *f* is IF strongly *β*-open mapping then *f*(*β*int⁡*V*) is an IF*β*OS in *Y* for IF*β*OS *V* in *X*. Hence *f*(*β*int⁡*V*) = *β* int*f*(*β*int⁡*V*) = *β*int⁡*f*(*V*). Thus *f*(*β*int⁡*V*)⊆*β*int⁡*f*(*V*).

Conversely, let *V* be IF*β*OS in *X* and then *V* = *β*int⁡*V*. Then by hypothesis, *f*(*V*) = *f*(*β*int⁡*V*)⊆*β*int⁡*f*(*V*). This implies that *f*(*V*) is IF*β*OS in *Y*.

**Theorem 41 .**

Let (*X*, *τ*) and (*Y*, *σ*) be IFTS and let *f* : (*X*, *τ*)→(*Y*, *σ*) be intuitionistic fuzzy *β*-weakly continuous, surjective mapping. If (*X*, *τ*) is IF *β*-compact space then (*Y*, *σ*) is IF *β*-almost compact.

**Proof**

Let {*A*_*i*_ : *i* ∈ *I*} be IF *β*-open cover of *Y* such that ∪_*i*∈*I*_ *A*_*i*_ = 1_~*Y*_. Then ∪_*i*∈*I*_ *f* ^−1^(*A*_*i*_) = *f* ^−1^(∪_*i*∈*I*_ *A*_*i*_) = *f* ^−1^(1_~*Y*_) = 1_~*X*_. (*X*, *τ*) is IF *β*-compact, and there exists a finite subset *I*_0_ of *I* such that ∪_*i*∈*I*_0__ *f* ^−1^(*A*_*i*_) = 1_~*X*_. Since *f* is IF *β*-weakly continuous, *f* ^−1^(*A*_*i*_)⊆*β*int⁡(*f* ^−1^(*β*cl⁡*A*_*i*_))⊆*f* ^−1^(*β*cl⁡*A*_*i*_). This implies that ∪_*i*∈*I*_0__ *f* ^−1^(*β*cl⁡*A*_*i*_)⊇∪_*i*∈*I*_0__ *f* ^−1^(*A*_*i*_) = 1_~*X*_. Thus ∪_*i*∈*I*_0__ *f* ^−1^(*β*cl⁡*A*_*i*_) = 1_~*X*_. Since *f* is surjective, 1_~*Y*_ = *f*(1_~*X*_) = *f*(∪_*i*∈*I*_0__ *f* ^−1^(*β*cl⁡*A*_*i*_)) = ∪_*i*∈*I*_0__ *f*(*f* ^−1^(*β*cl⁡*A*_*i*_)) = ∪_*i*∈*I*_0__ *β*cl⁡*A*_*i*_. Hence ∪_*i*∈*I*_0__ *β*cl⁡*A*_*i*_ = 1_~*Y*_. Hence (*Y*, *σ*) is IF *β*-almost compact.

**Theorem 42 .**

Let (*X*, *τ*) and (*Y*, *σ*) be intuitionistic fuzzy topological spaces and let *f* : (*X*, *τ*)→(*Y*, *σ*) be intuitionistic fuzzy *β*-irresolute, surjective, and strongly *β*-open mapping. If (*X*, *τ*) is IF *β*-nearly compact space then so is (*Y*, *σ*).

**Proof**

Let {*A*_*i*_ : *i* ∈ *I*} be any intuitionistic fuzzy *β*-open cover of (*Y*, *σ*). Since *f* is IF *β*-irresolute, then {*f* ^−1^(*A*_*i*_) : *i* ∈ *I*} is an intuitionistic fuzzy *β*-open cover of *X*. Since (*X*, *τ*) is IF *β*-nearly compact, there exists a finite subset *I*_0_ of *I* such that ∪_*i*∈*I*_0__ *β*int⁡(*β*cl⁡*f* ^−1^(*A*_*i*_)) = 1_~*X*_. Since *f* is surjective, 1_~*Y*_ = *f*(1_~*X*_) = *f*(∪_*i*∈*I*_0__ *β*int ⁡(*β*cl⁡*f* ^−1^(*A*_*i*_))) = ∪_*i*∈*I*_0__ *f*(*β* int⁡⁡⁡ (*β*cl⁡*f* ^−1^(*A*))). Since *f* is IF strongly *β*-open, *f*(*β*int⁡(*β*cl⁡*f* ^−1^(*A*_*i*_)))⊆*β*int⁡*f*(*β* cl*f* ^−1^(*A*_*i*_)) for each *i* ∈ *I*. Since *f* is IF *β*-irresolute, then *f*(*β*cl⁡*f* ^−1^(*A*_*i*_))⊆*β*cl⁡*f*(*f* ^−1^(*A*_*i*_)). Hence we have 1_~*Y*_ = ∪_*i*∈*I*_0__ *f*(*β*int⁡(*β*cl⁡*f* ^−1^(*A*_*i*_)))  ⊆  ∪_*i*∈*I*_0__ *β*int⁡*f*(*β*cl⁡*f* ^−1^(*A*_*i*_))  ⊆  ∪_*i*∈*I*_0__ *β*int⁡(*β*cl⁡*f*(*f* ^−1^(*A*_*i*_))) = ∪_*i*∈*I*_0__ *β*int⁡(*β*cl⁡(*A*_*i*_)). Thus 1_~*Y*_ = ∪_*i*∈*I*_0__ *β*int⁡(*β*cl⁡(*A*_*i*_)). Hence (*Y*, *σ*) is IF *β*-nearly compact.
